# NLRP3 augmented resistance to gemcitabine in triple-negative breast cancer cells via EMT/IL-1β/Wnt/β-catenin signaling pathway

**DOI:** 10.1042/BSR20200730

**Published:** 2020-07-03

**Authors:** Qiao Zheng, Dejiao Yao, Yi Cai, Tiecheng Zhou

**Affiliations:** 1 Department of Oncology, Hospital of Chengdu University of Traditional Chinese Medicine. No. 39 Twelve Bridges Road, Jinniu District, Chengdu 610072, China; 2 Department of Oncology, Sichuan Integrative Medicine Hospital. No. 51, Fourth Section of Renmin South Road, Chengdu 610041, China

**Keywords:** gemcitabine, IL-1β, NLRP3, resistance, Wnt/β-catenin

## Abstract

**Background:** Gemcitabine is widely used in the treatment of breast cancer (BC). However, the resistance to drugs remains a tough concern. The study explored the potential mechanism concerning gemcitabine resistance in triple-negative BC (TNBC) *in vitro*. **Methods:** TNBC cells (TNBCC) and gemcitabine-resistance cell lines (GRC) were used. We investigated the sensitivity to gemcitabine responsive to regulation of Nod-like receptor protein 3 (NLRP3) expression in TNBCC in different gemcitabine concentrations. RT-PCR checked NLRP3 mRNA expression and MTT assessed the cell cytotoxicity. Gemcitabine resistance was studied in GRC exposed to 0, 1, 3, 5 nm gemcitabine after GRC were treated with NLRP3 agonist Nigericin sodium salt (NSS) or antagonist CY-09. Epithelial-to-mesenchymal transition (EMT) biomarkers were evaluated via RT-PCR and inflammasome IL-1β, β-catenin content and GSK-3β activity were measured by ELISA methods. Last, we inactivated the signaling and examined the NLRP3, EMT mRNA expression by RT-PCR, IL-1β, β-catenin content and GSK-3β activity by ELISA and cell cytotoxicity through MTT. **Results:** NLRP3 up-regulation improved cell survival and reduced sensitivity to gemcitabine (*P*<0.05). NLRP3 had higher expression in GRC than TNBCC. GRC cell viability dropped as the gemcitabine concentration increased. NLRP3 up-regulation added to resistance to gemcitabine in GRC (*P*<0.05). NLRP3 agonist might induce EMT process, activate wnt/β-catenin signaling and IL-1β, while inactivation of wnt/β-catenin signaling could result in the inhibition of NLRP3, IL-1β and EMT as well as cell viability in GRC (*P*<0.05). **Conclusion:** NLRP3 could enhance resistance to gemcitabine via IL-1β/EMT/Wnt/β-catenin signaling, which suggested that NLRP3 antagonist CY-09 might be incorporated into gemcitabine treatment for TNBC.

## Introduction

Gemcitabine has been widely introduced to the treatments of cancers and has been proved as a curative drug alone or combined with other drugs, including cisplatin [1–3]. However, resistance to gemcitabine in cancer patients occurs commonly, which deters the curative effect [4]. Such situation hardly differs in breast cancer (BC). BC has high motality among all cancers and is highly invasive, especially triple-negative BC (TNBC) subtype [5]. In clinical practices, gemcitabine might exert better effect when used with other drugs like cisplatin or paclitaxel [6]. It was supported by a recent discovery based on clinical practice that gemcitabine functions better with cisplatin than with paclitaxel [7]. Nevertheless, due to the existence of drug resistance, the efficacy is discounted.

Previously, it was reported that mRNAs were involved with chemoresistance in BCs. PTEN could obstruct the resistance against gemcitabine and inhibited epithelial-to-mesenchymal transition (EMT) via AKT signaling [8]. However, previous researches have not provided sufficient proofs for therapeutic targets incorporated into chemotherapies, which require more intensive researches in the near future.

NLRP3, Nod-like receptor protein 3, belongs to the canonical inflammasome family of leucine-rich repeat containing proteins (NLRs) and mediates cancer pathogenesis via mediation of apoptosis proteins and immune responses [9,10]. Also, it is reported that NLRP3 is activated by chemotherapy in human tumors [11]. Activation of NLRP3 could induce the IL-1β and IL-22 to advance lung cancer metastasis [12]. Besides, previous research suggested that NLRP3 could induce drug resistance in the treatment of acute lymphoblastic leukemia [13]. In BC cells, it was previously displayed that the depletion of NLRP3 could regulate cell viability [14]. Furthermore, clinical analysis also showed that NLRP3 expression was correlated with BC metastasis and lymphogenesis [12]. Whereas, there are few researches that have explored the role of NLRP3 in resistance to chemotherapy in BC, neither *in vitro* nor *in vivo*.

BC is highly invasive and *in vitro* it presents high EMT process, which could be manifested through changes in expression of EMT-gain biomarkers N-cadherin, Vimentin and EMT-loss biomarker E-cadherin. Previous research concluded that Wnt/β-catenin signaling pathway was significant for regulation of metastasis, cell proliferation and EMT in BC even though the localization of β-catenin in cancer cells differed depending on the specific types of BC [15]. In the subtype, TNBCs, wnt signaling gene GSK3β regulated EMT, suggesting that GSK3β might be a promising target in suppressing TNBC in clinical stage [16]. Researchers currently discovered that the proper combination of Paclitaxel and Wnt signaling inhibitor could effectively enhance the cell cytotoxicity in TNBCs, which prevented metastasis via inactivation of Wnt/β-catenin signaling pathway [17]. Based on the background introduced above, we hypothesized that NLRP3 might be associated with gemcitabine resistance in TNBC through mediation of Wnt/β-catenin signaling pathway.

Therefore, in the present study, we investigated the regulatory role of NLRP3 in resistance to gemcitabine in TNBCs and its underlying interaction with Wnt/β-catenin *in vitro*.

## Methods

### Cell culture and treatment

TNBC cell lines (TNBCC), MDA-MB-231 and MDA-MB-468 were purchased (ATCC, CA, U.S.A.). The gemcitabine-resistance cell lines (GRC), MDA-MB-231-R and MDA-MB-468-R were produced by treating TNBCC with increasing gemcitabine concentrations (0.1–15 nm) for 12 months (GI50 < 7.0) [18]. Gemcitabine was bought from AbMole (Beijing, China). Cells were cultured in DMEM (PM150210) containing 4500 mg/l glucose, 10% fine FBS (164220-100) and 100 U/ml penicillin–streptomycin (PB180122) in 5% CO_2_, 95% O_2_ condition at 37°C (Procell, Wuhan, China).

Each cell line was also incubated with NLRP3 agonist Nigericin sodium salt (NSS) and antagonist CY-09 separately (Selleck, Shanghai, China) to differentiate NLRP3 expression in both TNBCC and GRC. Wnt inhibitor Wnt-C59 was added into subgroups of GRC so as to inactivate signaling pathway (Selleck, Shanghai, China).

All the cell lines were exposed to the 0, 1, 3, 5 nM gemcitabine, respectively, for 72 h for the following assays.

### RT-PCR

The total RNA was extracted from cells with different drug treatments by TRIzol based on the guidebook of the producer (Beyotime, Shanghai, China). IScript Advanced cDNA synthesis kit was applied to synthesize cDNA (Bio-Rad, CA, U.S.A.). Cells were selected and planted into 96-well plates for RT-PCR assays. SYBR Green qPCR Master Mix (MedChemExpress, Shanghai, China) was adopted for PCR analysis. The sequences for the primers were listed as below. NLRP3: F, 5′-ACTCTGTGAGGGACTCTTGC-3′; R, 5′-GGTCGCCCAGGTCATTGTT-3′ [19]. E-cadherin: F, 5′-CGGGAATGCAGTTGAGGATC-3′; R, 5′-AGGATGGTGTAAGCGATGGC-3′. Vimentin: F, 5′-ACGTCTTGACCTTGAACGCA-3′; R, 5′-TCTTGGCAGCCACACTTTCA-3′ [20]. GAPDH: F, 5′-GAGCCCGCAGCCTCCCGCTT-3′; R, 5′-CCCGCGGCCATCACGCCACAG-3′ [21]. The PCR values were calculated in 2^−ΔΔ*C*_t_^ method.

### ELISAs for IL-1β, GSK-3β and β-catenin activity

The cells from MDA-MB-231, MDA-MB-468, MDA-MB-231-R, MDA-MB-468-R, MDA-MB-231-R CY-09, MDA-MB-468-R CY-09, MDA-MB-231-R Wnt-C9, MDA-MB-468-R Wnt-C9 groups were collected and cultured till they reached ∼80% confluence. Then lysis buffer in the ELISA Kits mentioned below were added to lyse the mixture. BCA Assay Kit was utilized to quantify the protein in each group. A total of 10 μg protein extracts of all groups were used for each of the following ELISA methods based on the manufacturer’s instructions. IL-1β ELISA Kit was used according to the provided instructions from the producer to measure the IL-1β cytokine activity in each group mentioned above (Thermo Fisher, Beijing, China). GSK3β activity was evaluated using GSK-3β (pS9) + Total GSK-3β Simple Step ELISA Kit strictly guided by the instructions on the guidebook (Abcam, Beijing, China). β-catenin accumulation was detected using β-catenin ELISA Kit (Thermo Fisher, Beijing, China).

### MTT assay for cell cytotoxicity

Cells were seeded into 96-well plates with 2800 cells each well and the plates were incubated in the incubator with 5% CO_2_ at 37°C for a day. Then cells were exposed to different gemcitabine concentrations (0, 1, 3, 5 nm). After 48-h exposure, we added 10 μl MTT (5 mg/ml) solution into each well and left the plates to incubate inside the incubator for another 4 h. Thereafter, 100 μl Formazan solution was put in each well and the plates were in incubation for another 4 h. Absorbance values were evaluated at 570 nm.

### Statistical analysis

All the assays were performed for three times in independence. SPSS Statistical 19 (IBM, Seattle, U.S.A.) was applied for *P*-values, double-tailed Student’s *t* test. All the data were shown in figures as mean and standard deviation values. GraphPad Prism 8 (GraphPad, CA, U.S.A.) contributed to the formation of figures in tiff format based on the raw data.

## Results

### Up-regulated NLRP3 promoted resistance to gemcitabine in TNBC *in vitro*

**Figure 1:**
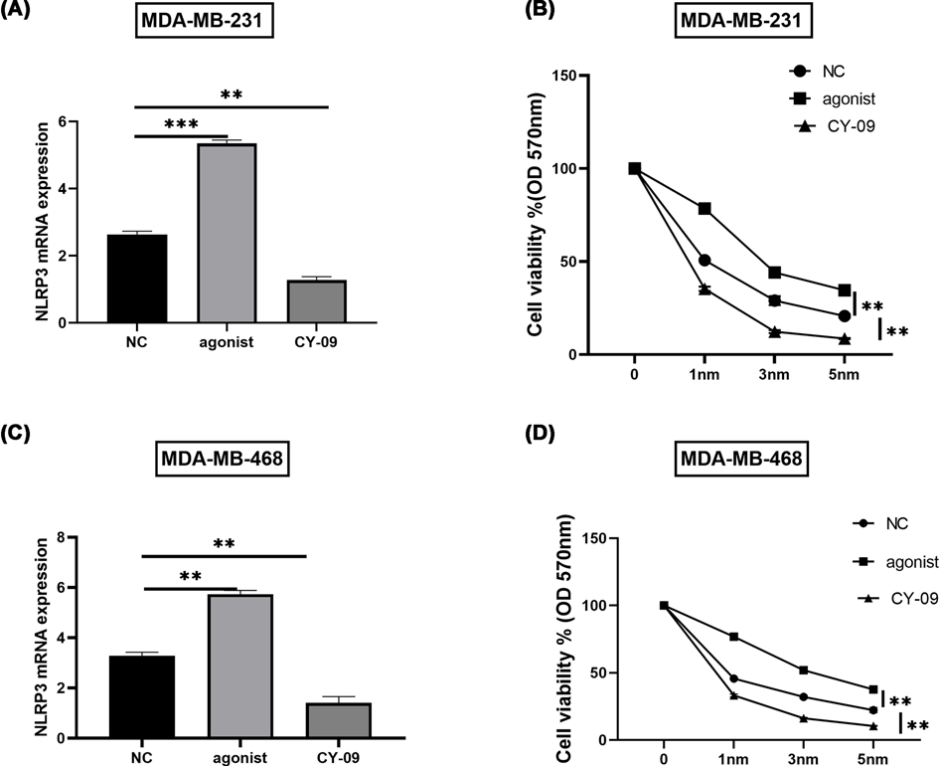
Up-regulated NLRP3 reduced sensitivity to gemcitabine in TNBC *in vitro* TNBC cell lines, MDA-MB-231 and MDA-MB-468 were used. MDA-MB-231 and MDA-MB-468 cell lines were treated with NLRP3 agonist NSS or antagonist with the non-treated cell lines as controls. (**A,C**) RT-PCR assessed relative mRNA expression of NLRP3 by 2^−ΔΔ*C*_q_^ method. (**B,D**) After regulation of NLRP3 in both cell lines, the cells in all the three groups were exposed to 0, 1, 3, 5 nm gemcitabine for 72 consecutive hours. MTT detected the cytotoxicity in different groups after exposure to different concentrations of gemcitabine. OD value was measured at 570 nm. All the assays were performed for three times independently. Average and SD values were presented; ***, *P*<0.01;**, *P*<0.05.

In both the TNBC cells, NLRP3 was either inhibited by its antagonist CY-09 or agonized through its agonist NSS. RT-PCR measured the mRNA expression and observed an increase in agonist group and a decrease in CY-09 group, using a non-treatment group as a control (*P*<0.05, Figure 1A,C). MTT assessed the cell cytotoxicity in three groups with exposure to different concentrations of gemcitabine (0, 1, 3, 5 nm). The linear graph presented the cytotoxicity at 72 h when exposed to different concentrations. It showed that highest cell viability was seen in agonist group with higher NLRP3 and lowest in antagonist group, suggesting that the higher expression of NLRP3, the less sensitive to different gemcitabine doses (*P*<0.05, Figure 1B,D).

### Inhibition of NLRP3 alleviated the gemcitabine resistance in TNBC *in vitro*

**Figure 2:**
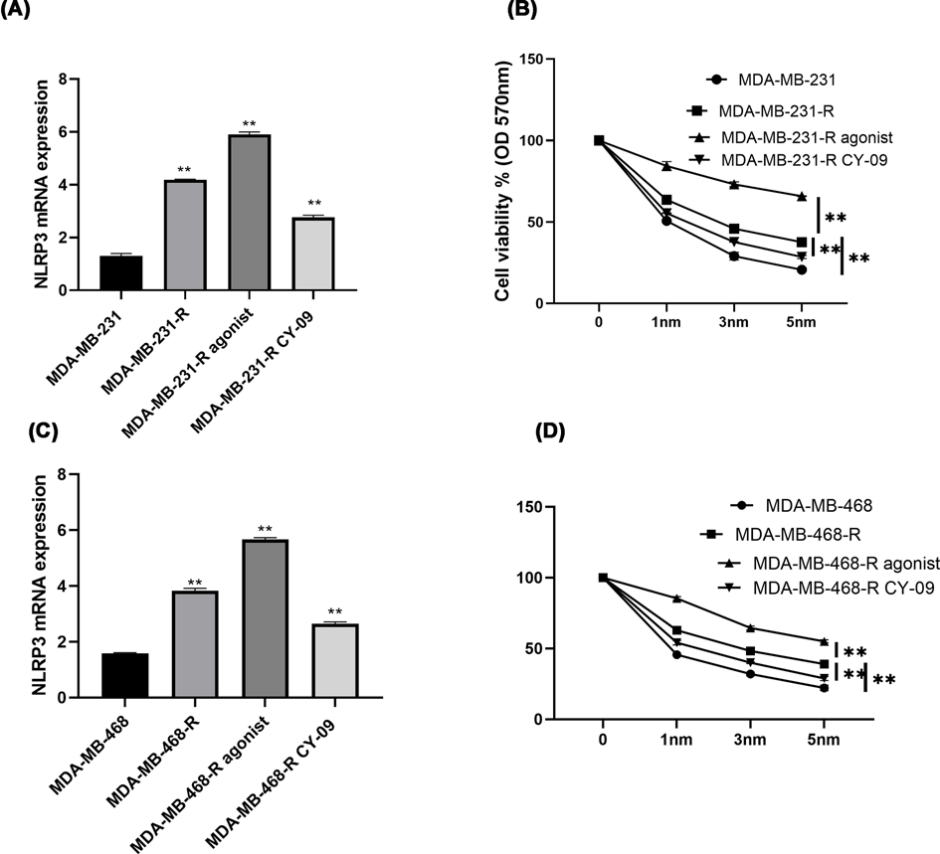
Inhibition of NLRP3 alleviated the gemcitabine resistance in TNBC *in vitro* The GRCs were developed by exposing the TNBC cell lines to increasing gemcitabine treatments (0.1–15 nm) for a year. GRC divided by incubation with NLRP3 agonist NSS or antagonist CY-09 with non-treated cells as baselines. The normal TNBC cell lines were also involved here as a comparison. (**A,C**) RT-PCR checked the relative mRNA expression NLRP3 in the four groups, TNBC cell line, respective GRC, GRC+ agonist and GRC+CY-09. (**B,D**) All the groups were exposed to 0, 1, 3, 5 nm gemcitabine for 72 h. MTT observed the cytotoxicity accordingly. OD value was measured at 570 nm. All the assays were performed thrice, based on which, Mean and SD values were obtained and presented in the figures. **, *P*<0.05.

First, differential mRNA expression of NLRP3 was measured in TNBCC, GRC, GRC treated with NSS and GRC treated with CY-09 groups, and it was discovered that NLRP3 had higher expression in GRC than in normal BC cells (TNBCC) (*P*<0.05, Figure 2A,C). It also showed a rise in NSS group and a fall in CY-09 treated GRC with GRC group as a baseline (*P*<0.05, Figure 2A,C). Next, the cytotoxicity in each group was monitored with MTT when the cells were treated with 0, 1, 3 and 5 nm gemcitabine and findings tended to prove that agonized NLRP3 could better resist the inhibitory effect of growing gemcitabine, in other words, higher expression of NLRP3 decreased the cytotoxicity and added to the gemcitabine resistance while inhibition of NLRP3 increased cytotoxicity in TNBCC, helping to alleviate the drug resistance (*P*<0.05, Figure 2B,D).

### NLRP3 antagonist inhibited EMT, IL-1β and Wnt/β-catenin signaling pathway in gemcitabine-resistant TNBC *in vitro*

**Figure 3:**
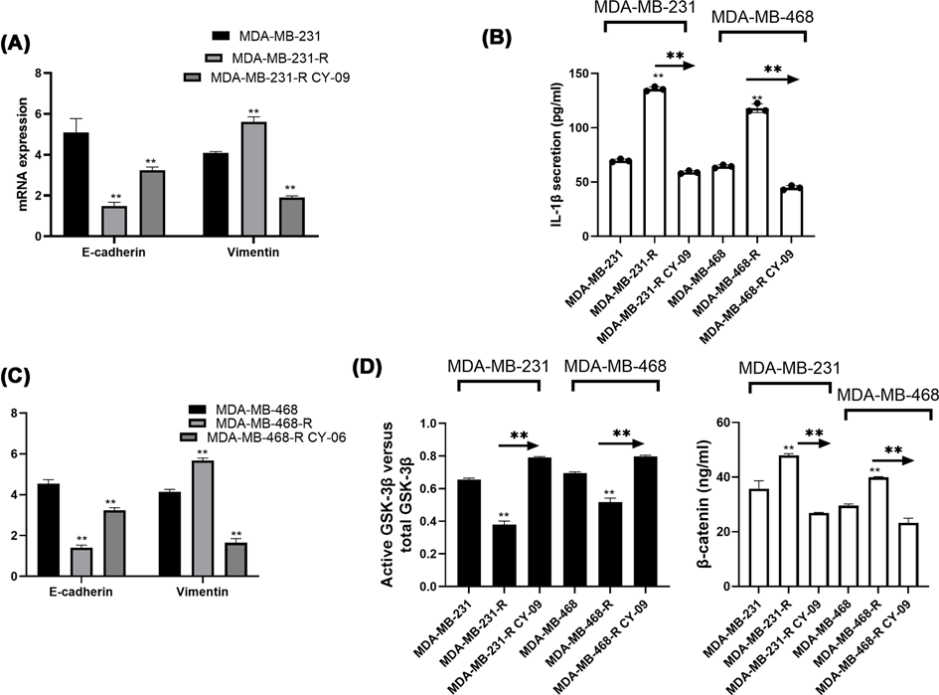
NLRP3 antagonist inhibited EMT, IL-1β and Wnt/β-catenin signaling pathway in gemcitabine-resistant TNBC *in vitro* Three groups were divided here, namely, TNBC cell line, respective GRC line and GRC treated with NLRP3 inhibitor CY-09. (**A,C**) RT-PCR evaluated the relative mRNA expression of E-cadherin and Vimentin in all the groups. (**B,D**) ELISA methods measured the IL-1β secretion, p-GSK3β-Ser^9^ and β-catenin protein expression so as to monitor the activity of Wnt/β-catenin signaling pathway. All the assays were performed thrice. **, *P*<0.05.

RT-PCR observed higher Vimentin and lower E-cadherin in GRC than TNBCC, suggesting that EMT process was promoted in gemcitabine-resistance cells (*P*<0.05, Figure 3A). However, NLRP3 antagonist CY-09 inhibited EMT process in GRC (*P*<0.05, Figure 3A,C). ELISA results displayed that inflammatory cytokine IL-1β was higher in resistant cells and inhibited by NLRP3 down-regulation (*P*<0.05, Figure 3B). In addition, GSK3β activity was reduced and total β-catenin content was enhanced in GRC compared with normal TNBCC and partly counteracted when incubated with CY-09, indicating the inactivation in Wnt/β-catenin signaling by NLRP3 inhibitor (*P*<0.05, Figure 3D). The details of the GSK3β activity in each group were displayed in the Supplementary Tables S1 and S2. The three repeated results were included.

### Inactivation of Wnt/β-catenin signaling inhibited NLRP3, IL-1β, EMT and cell viability

**Figure 4:**
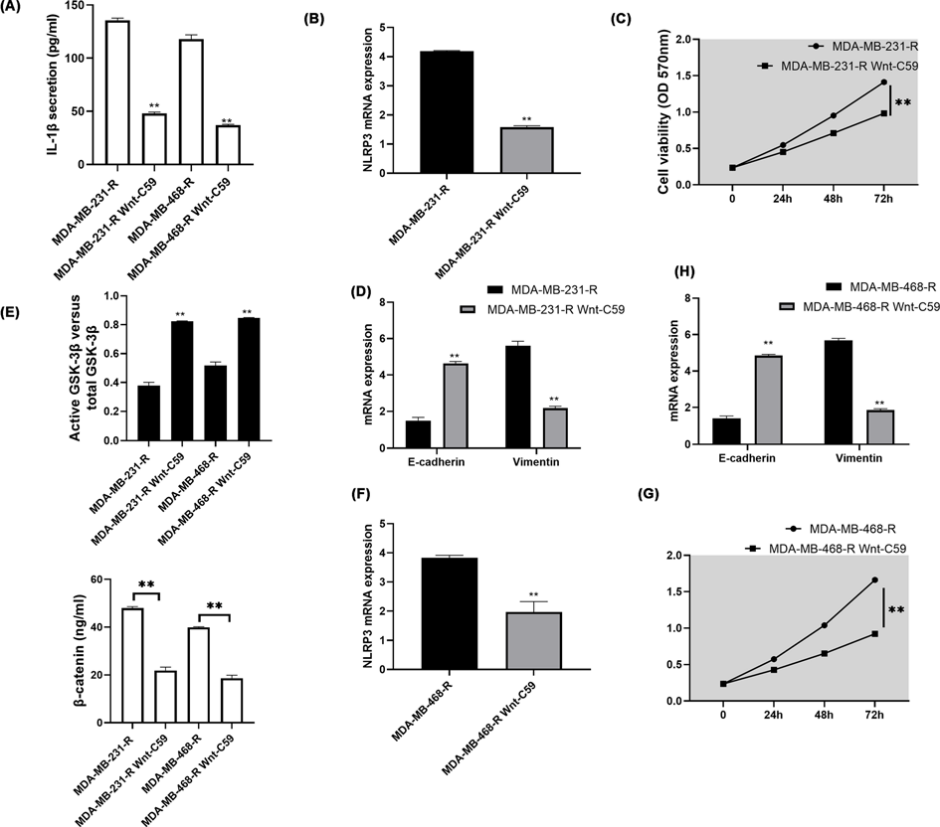
Inactivation of Wnt/β-catenin signaling inhibited NLRP3, IL-1β, EMT and cell viability The GRC cell lines were split into two subtypes, with one incubated with an inhibitor of Wnt/β-catenin signaling, Wnt-C59 and the other normal GRC as a control. (**A,E**) ELISA examined the activity of GSK3β and β-catenin so as to ensure the inhibitory effect of Wnt-C59 and IL-1β secretion was assessed thereafter. (**B,F**) RT-PCR verified the NLRP3 expression after signaling inactivation. (**C,G**) MTT measured cell viability. (**D,H**) RT-PCR finally observed the relative mRNA expression of E-cadherin and vimentin with normalization to GAPDH. All the assays were performed thrice. **, *P*<0.05.

ELISA confirmed that GSK3β activity was up-regulated and β-catenin content was inhibited in the GRC incubated with Wnt-C59 (*P*<0.05, Figure 4E). Besides, ELISA results showed that IL-1β cytokine was suppressed by Wnt-C59 (*P*<0.05, Figure 4A). RT-PCR presented lower NLRP3, Vimentin and higher E-cadherin mRNA expression when cells were treated with Wnt-C59, indicating lower EMT process in GRC with inactivated Wnt/β-catenin signaling (*P*<0.05, Figure 4B,D,F,H). Furthermore, MTT suggested lower cell viability when the signaling was inactivated (*P*<0.05, Figure 4C,G).

## Discussion

BC is mainly constituted of three subtypes, HR positive, human epidermal growth factor (HER2)-enriched and TNBC according to immunohistochemical expression of the hormone receptors (HR): estrogen receptor (ER) and progesterone receptor (PR) as well as HER2 [22]. TNBC presents absence of HR and up-regulated HER2 and has highest metastasis among the three subtypes of BCs [23]. For patients with TNBC, chemotherapy is a good option, which might help to lead to a better prognosis [24]. Yet, single drug-targeted therapy proved relatively ineffective due to frequent occurrences of chemotherapy resistance in TNBC treatment [25]. In recent years, signaling pathway targeted therapy drew great interest from researchers, among which, PTEN, Pi3k/Akt/mTOR and JAK/STAT signaling pathways presented promising potentials [26]. Later on, previous findings disclosed better efficacy in the combined therapy with drugs and signaling mediators [26]. Though, neoadjuvant chemotherapies including traditional drug combination or a mix of drugs and gene targets, improved the curative effect of BC, the drug resistance still widely exists, which requires more investigations into the molecular mechanisms that deters drug sensitivity [27]. Therefore, we explored potential therapeutic targets in Gemcitabine-resistant TNBC cell lines in hope that the finding could enrich the knowledge of regulatory mechanisms beneath drug resistance in gemcitabine treatments of TNBC.

Researchers unveiled that NLRP3 stimulation could mediate the resistance to 5-Fluorouracil both *in vitro* and *in vivo* in oral squamous cell carcinoma [28]. Besides, NLRP3, the canonical inflammasome, was disclosed to be aberrant in TNBC [29]. However, there has not been much reporting about the connections between gemcitabine resistance and NLRP3 in TNBC. In the current research, we hypothesized that NLRP3 might be involved in the resistance to gemcitabine in TNBC. To simplify, we selected two different TNBC cell lines (TNBCC) and established respective Gemcitabine-resistant TNBC cells (GRC). Then, we up-regulated and inhibited NLRP3 in the cell lines using its agonist NSS and antagonist CY-09. After confirmation of the up- or down-regulation of NLRP3 in TNBCC by RT-PCR assays, we found that gemcitabine cytotoxicity decreased in up-regulated NLRP3 group and as gemcitabine concentration increased, the cytotoxicity enhanced at a decreasing rate, indicating that NLRP3 agonist could enhanced the gemcitabine resistance. On the other hand, the antagonist CY-09 brought lower expression of NLRP3, inducing the increased cytotoxicity, thereby suggesting the inhibition of resistance against gemcitabine in TNBCC. The findings support that NLRP3 mediates gemcitabine-resistance in TNBC cellular model. Besides, RT-PCR showed higher NLRP3 expression in GRC, suggesting that gemcitabine activates NLRP3, which is in accordance with previous discovery in other tumors [11].

Recently NLRP3 was exposed to play a role in regulation of BC metastasis by activation of IL-1β [30]. Besides, IL-1β expression was highly correlated with metastasis in TNBC and *in vitro*, increased IL-1β induced higher invasiveness of TNBC cells [31]. Thus, we investigated the respective IL-1β activity in response to NLRP3 regulation, which showed that up-regulation of NLRP3 elevated IL-1β activity in TNBC cells. As widely recognized, cell migratory and invasive properties can be manifested by the EMT process [32]. Furthermore, we examined the EMT process through detection of the epithelial gene E-cadherin, and mesenchymal gene Vimentin by RT-PCR and found decreased E-cadherin and increased Vimentin in the NLRP3 agonist group, supporting that NLRP3 promoted the EMT process in Gemcitabine-resistant cells in TNBC perhaps by inducing IL-1β secretion.

EMT and Wnt/β-catenin signaling regulate the sensitivity to chemodrugs in TNBC [33,34]. GSK3β activity degrades β-catenin, thus silencing the Wnt/β-catenin signaling [35]. The phosphorylation of GSK3β can occur at Ser^9^ and Tyr^216^. Phospho-GSK3β-Ser^9^ suppresses GSK3β activity, thereby inducing the β-catenin content while Phospho-GSK3β-Tyr^216^ promotes the activity and inhibits the β-catenin [36]. In our study, we detected the p-GSK3β-Ser^9^, total GSK3β and β-catenin levels by ELISA and calculated the GSK3β activity accordingly in TNBC, GRC, inhibited NLRP3 and signaling blocked groups (Supplementary Tables S1 and S2). Meanwhile, we also evaluated the β-catenin content by ELISA method. Our results present that the lower p-GSK3β-Ser^9^ induced by NLRP3 inhibitor CY-09 or signaling suppressor Wnt-C59 indicates higher GSK3β activity and suppresses β-catenin. Taken together, we assessed the potential influence of NLRP3 expression on IL-1β/EMT and Wnt/β-catenin signaling and and discovered that higher NLRP3 expression resulted in more active IL-1β, EMT process and Wnt/β-catenin signaling.

In TNBC, wnt3A inhibitor was revealed to slow down EMT process with a hint that Wnt/β-catenin signaling could modulate EMT [37]. Hence, in our study, we inhibited Wnt/β-catenin signaling with Wnt-C59 and then researched the changes in NLRP3, cell viability and IL-1β in gemcitabine-resistance cells, which led us to the discovery that inactivation of Wnt/β-catenin signaling inhibited NLRP3 expression and IL-1β secretion, deterred EMT process and suppressed cell viability in GRC, suggesting that the inhibition in Wnt/β-catenin signaling reduced the cellular resistance to gemcitabine by suppressing NLRP3, curbing IL-1β and EMT process in TNBC, verifying our conjecture that NLRP3 modulates the gemcitabine-resistance through IL-1β/EMT/Wnt /β-catenin signaling in TNBC *in vitro*.

## Conclusion

The study presented the *in-vitro* findings that NLRP3 down-regulation could increase the gemcitabine sensitivity in TNBC cells and that in gemcitabine-resistant cells, NLRP3 up-regulation could enhance the drug resistance via activation of IL-1β, EMT and Wnt/β-catenin signaling, which posed a potential possibility that NLRP3 antagonist could be a therapeutic strategy that can be incorporated into gemcitabine treatment to promote the efficacy by reducing the drug resistance in TNBC in the future.

## Highlights

1. The current research features the contribution of NLRP3 to gemcitabine resistance in TNBCC.
2. It was disclosed that up-regulated NLRP3 reduced sensitivity to gemcitabine in TNBC cells and inhibition of NLRP3 alleviated the gemcitabine resistance in TNBC *in vitro.*
3. NLRP3 antagonist inhibited NLRP3 expression, resulting in the suppression of cell viability, EMT process and IL-1β through inactivation of Wnt/β-catenin signaling pathway in gemcitabine-resistant TNBC *in vitro.*
4. Inactivation of Wnt/β-catenin signaling inhibited NLRP3 expression, deterring IL-1β, EMT and cell viability of GRC in gemcitabine-resistant TNBC.

## Supplementary Material

Supplementary Tables S1-S2

## Abbreviations

BC: breast cancer
EMT: epithelial-to-mesenchymal transition
GI50: growth inhibitory 50%
GRC: gemcitabine-resistance cell line
GSK-3β: glycogen synthase kinase 3 beta
HER2: human epidermal growth factor
HR: hormone receptor
IL: interleukin
MTT: 3-(4,5-dimethyl-2thiazolyl)-2,5-diphenyl-2H-tetrazolium bromide
NLRP3: Nod-like receptor protein 3
NSS: Nigericin sodium salt
TNBC: triple-negative BC
TNBCC: TNBC cell
