# Supplementary material for: NLRP3 augmented resistance to gemcitabine in triple-negative breast cancer cells via EMT/IL-1β/Wnt/β-catenin signaling pathway
Source: Biosci Rep. 2020 Jul 3;40(7):BSR20200730. doi: 10.1042/BSR20200730 (PMC7335831; doi:10.1042/BSR20200730)
Supplement: Supplementary Tables S1-S2 [file BSR-2020-0730_supp.pdf]

Table 1. Details of GSK-3 beta activity in MDA-MB-231 cells after different treatments

	MDA-MB-231	MDA-MB-231-R	MDA-MB-231-R CY-09	MDA-MB-231-R Wnt-C59
P-GSK-3β(Ser9) ug/ml	70.287	123.832	41.602	36.474
	72.766	126.403	42.671	37.67
	74.509	122.221	43.479	38.620
GSK-3β (ug/ml)	210.123	201.357	199.373	210.122
	206.263	203.254	209.033	213.781
	215.246	195.357	201.937	211.569
P-GSK-3β(Ser9)/ GSK-3β	0.335	0.607	0.208	0.174
	0.353	0.609	0.204	0.176
	0.346	0.647	0.215	0.182
Active GSK-3β/GSK-3β	0.665	0.393	0.792	0.826
	0.647	0.391	0.796	0.824
	0.654	0.353	0.785	0.818

Table 2. Details of GSK-3 beta activity in MDA-MB-468 cells after different treatments

	MDA-MB-468	MDA-MB-468-R	MDA-MB-468-R CY-09	MDA-MB-468-R Wnt-C59
P-GSK-3β(Ser9) ug/ml	61.414	99.982	41.691	32.627
	58.689	97.898	38.534	32.216
	63.770	105.084	40.278	31.563
GSK-3β (ug/ml)	201.121	210.082	198.761	209.182
	198.032	211.877	196.893	208.085
	205.031	205.792	199.539	212.227
P-GSK-3β(Ser9)/ GSK-3β	0.305	0.476	0.210	0.156
	0.296	0.462	0.196	0.155
	0.311	0.511	0.202	0.149
Active GSK-3β/GSK-3β	0.695	0.524	0.790	0.844
	0.704	0.538	0.804	0.845
	0.689	0.489	0.798	0.851
